# Effectiveness of dog collars impregnated with 4% deltamethrin in controlling visceral leishmaniasis in *Lutzomyia longipalpis* (Diptera: Psychodidade: Phlebotominae) populations

**DOI:** 10.1590/0074-02760170377

**Published:** 2018-03-26

**Authors:** Rafaella Albuquerque e Silva, Andrey José de Andrade, Bruno Beust Quint, Gabriel Elias Salmen Raffoul, Guilherme Loureiro Werneck, Elizabeth Ferreira Rangel, Gustavo Adolfo Sierra Romero

**Affiliations:** 1 Universidade de Brasília, Núcleo de Medicina Tropical, Brasília, DF, Brasil; 2 Ministério da Saúde, Secretaria de Vigilância em Saúde, Brasília, DF, Brasil; 3 Universidade Federal do Paraná, Departamento de Patologia Básica, Curitiba, PR, Brasil; 4 Universidade Federal do Rio de Janeiro, Instituto de Medicina Social, Rio de Janeiro, RJ, Brasil; 5 Fundação Oswaldo Cruz-Fiocruz, Instituto Oswaldo Cruz, Rio de Janeiro, RJ, Brasil

**Keywords:** visceral leishmaniasis, Lutzomyia longipalpis, impregnated collars, deltamethrin, dogs

## Abstract

**BACKGROUND:**

There is little information on the effect of using deltamethrin-impregnated dog collars for the control of canine visceral leishmaniasis.

**OBJECTIVES:**

The objective of this study was to evaluate the effectiveness of the use of 4% deltamethrin-impregnated collars (Scalibor®) in controlling visceral leishmaniasis in *Lutzomyia longipalpis* by comparing populations in intervention and non-intervention areas.

**METHODS:**

Phlebotomine flies were captured over 30 months in four neighbourhoods with intense visceral leishmaniasis transmission in Fortaleza and Montes Claros. We calculated the rates of domicile infestation, relative abundance of *Lu. longipalpis*, and *Lu. longipalpis* distribution in each site, capture location (intra- and peridomestic locations) and area (intervention and non-intervention areas).

**FINDINGS:**

In the control area in Fortaleza, the relative abundance of *Lu. longipalpis* was 415 specimens at each capture site, whereas in the intervention area it was 159.25; in Montes Claros, the relative abundance was 5,660 specimens per capture site in the control area, whereas in the intervention area it was 2,499.4. The use of dog collars was associated with a reduction in captured insects of 15% (p = 0.004) and 60% (p < 0.001) in Montes Claros and Fortaleza, respectively.

**MAIN CONCLUSIONS:**

We observed a lower vector abundance in the intervention areas, suggesting an effect of the insecticide-impregnated collars.

Leishmaniasis is a neglected disease that requires special government attention for surveillance and control. Visceral leishmaniasis (VL) is endemic in 70 countries on five continents and is mainly found in South Asia, East Africa, and the Americas. Among South America countries, Brazil accounts for the largest number of cases, registering over 90% of cases notified (OPAS/WHO 2017). In the Americas, VL is a zoonosis caused by the protozoa *Leishmania infantum*, and it is a serious public health issue.

VL surveillance and control policies in Brazil, outlined by the Leishmaniasis National Program of the Brazilian Ministry of Health, are focused on three axes for specific activities. First, the use of chemical insecticides and environmental management are recommended to lower the vector population density and reduce vector-human contact. Second, canine serological surveys and adequate management of positive cases are endorsed to decrease the sources of infection for the vector. Finally, timely diagnosis and proper management of human cases are needed to prevent severe forms of the disease and death (MS/SVS/DVE 2014).

However, over the years, difficulties in the operationalisation of VL surveillance and control activities have been observed. The lack of effectiveness results partially from the shortage of qualified professionals and scarce financial resources. Moreover, delays in collections, performance of routine diagnostic tests, and removal of seropositive dogs, as well as refusal by dog owners to comply with surveillance measures, are frequent.

Mathematical models have suggested that euthanasia of *Leishmania* antibody-positive dogs in areas with low or moderate transmission of the pathogen might reduce the prevalence of canine infection in the long term (Costa et al. 2013). The infectiousness of asymptomatic animals, as well as their proportion among the whole dog population, can impact control strategies. Nevertheless, the indiscriminate euthanasia of asymptomatic dogs may compromise maintenance of the program because of population dissatisfaction.

Furthermore, in situations where seropositive dogs are removed, the rate of dog replacement is high (von Zuben and Donalísio 2016). In studies carried out in the city of Araçatuba (São Paulo state), the replacement rate was 44.5%, with owners justifying this by the need for a companion or guard dog for residencies (Andrade et al. 2007).

Regarding the vector, the ability of *Lutzomyia longipalpis* to adapt to urban environments increases the complexity of chemical control, resulting in operational difficulties, high costs, toxicity, and limited residual effects of the insecticide, and reducing the feasibility of executing this activity in these settings (von Zuben and Donalísio 2016). In addition, the lack of knowledge about the biology of immature forms of sandflies, mainly their breeding sites, is a key factor in the failure to control these insects.

Thus, alternatives to complement current Ministry of Health control strategies are highly desirable. Dog collars impregnated with 4% deltamethrin are considered as a potential tool for the control of canine VL. They contain a repellent and show insecticidal activities, reducing the interactions between dogs and phlebotomine flies. The efficacy of these collars has been described previously (David et al. 2001, Maroli et al. 2001, Gavgani et al. 2002, Reithinger et al. 2004). Killick-Kendrick et al. (1997) evaluated the insect repellency and insecticidal potential of collars impregnated with deltamethrin using *Phlebotomus perniciosus* in the laboratory. The dogs were followed up for 8 months and were periodically exposed to 200 *P. perniciosus* for two hours at least seven times between the 2nd and 34th week after the placement of the collars. An evaluation of repellency and mortality, based on the percentage of engorged and dead females after exposure, respectively, was conducted. The use of the collars prevented about 96% of *P. perniciosus* females from feeding during the 34 months of the study. Mortality of the exposed flies fluctuated between 21 and 60% during the period. A similar study was performed by David et al. (2001); however, they used the main vector associated with VL in the Americas, *Lu. longipalpis*. Feed interruption was demonstrated in 96% of flies used in the experiment, and fly mortality ranged from 90 to 35% over the period.

Once efficacy was demonstrated in the laboratory, effectiveness studies were performed to evaluate collars impregnated with 4% deltamethrin as a control tool for use in governmental programs. Based on the performance of collars in field trials, interventional studies were then conducted. These demonstrated a reduction in the prevalence of VL in treated dogs. Brazuna (2012) evaluated the reduction of disease prevalence in dogs after the use of Scalibor® collars in all dogs in the municipality of Campo Grande, MS. The reduction in disease prevalence in dogs was 50%. Comparable results were reported by Kazimoto, who showed a 53% prevalence reduction with the use of these collars in a smaller number of dogs. In addition, a mathematical modelling study performed by Sevá et al. (2016) indicated that the use of insecticide-impregnated collars, when 90% coverage is achieved, can decrease the prevalence of seropositive dogs and incidence of human VL cases to zero.

Nonetheless, it is necessary to evaluate the impact of using 4% deltamethrin-impregnated collars (Scalibor®) of populations of *Lu. longipalpis*, since proof of the insecticide or repellent effect of this intervention dogs does not necessarily equate with an effect at the population level (Gidwani et al. 2011). Therefore, the objective of this study was to evaluate the impact of this intervention by comparing populations of *Lu. longipalpis* in treated and untreated areas.

## MATERIALS AND METHODS

*Study area* - The study was carried out in Montes Claros, MG, and Fortaleza, CE. These municipalities were chosen because they were participating in the project entitled “Evaluation of the effectiveness of 4% deltamethrin-impregnated collars in endemic areas for visceral leishmaniasis”, a project commissioned by the Ministry of Health whose objective was to evaluate the impact of these collars on VL prevalence in dogs and incidence in humans. The project, however, did not evaluate the impact of these collars on *Lu. longipalpis* populations.

The areas in which dog collars were used (intervention areas) and were not used (control areas) were defined in the previous study. Inclusion of intervention and control areas in this study was based on the number of VL cases (only municipalities with an average of more than 4.4 cases in the last three years were included) and availability of teams to carry out entomological surveillance activities.

**Fig. 1:**
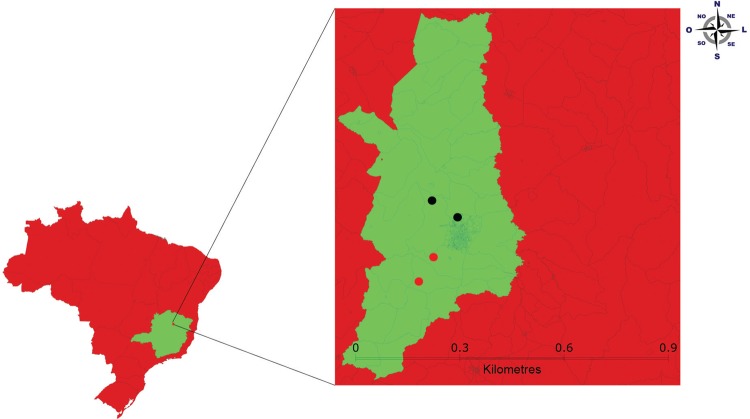
: Montes Claros municipality, Minas Gerais (MG), Brazil. Black dots indicate the control area and red dots indicate the intervention area.

**Fig. 2:**
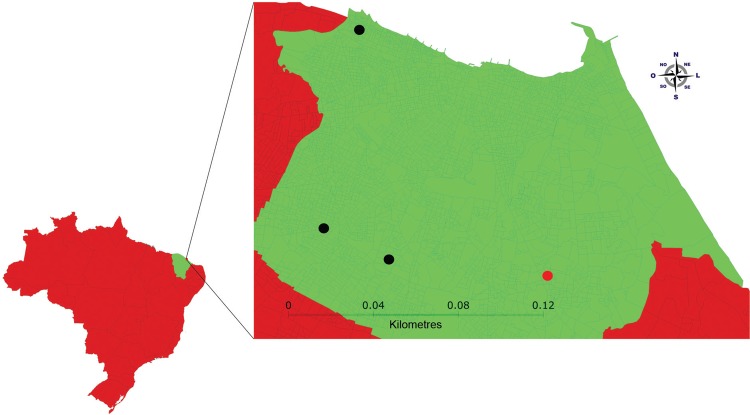
: Fortaleza municipality, Ceará (CE), Brazil. Black dots indicate the control area and red dots indicate the intervention area.

Montes Claros, with an area of 3,582.034 km^2^ and a population of 385,898 people, is located in the south-eastern region of Brazil (Fig. 1). Its vegetation comprises a mixture of *cerrado* and *caatinga*. It has a tropical climate with an average annual temperature of 22ºC, dry and mild (rarely excessively cold) winters, and hot rainy summers (Michalshy et al. 2009). Fortaleza is located in the north-eastern region of Brazil and has an average altitude of 21 metres, an area of 313.8 km^2^, and a population of 2,551,806 people. It is the state capital with the highest density (7,815.7 pop/km) of people in the country (Fig. 2). It has typical coastal vegetation with areas of mangrove and *restinga*. Its climate is tropical and semi-humid, with an annual average temperature of 26ºC. December and January are the hottest months of the year and July the coldest; however, the difference between these seasonal temperatures is minimal (Silva et al. 2014). Rainfall data from both municipalities during the study period were obtained through the National Institute of Meteorology website (INMET 2017).

In the intervention areas, the collars were placed on domestic or semi-domestic dogs. Stray dogs were not included in the intervention. In total, around 5,000 dogs per municipality wore the collars, which were replaced every six months. The 4% deltamethrin-impregnated collar used in this study, Scalibor®, is commercially available in two presentations: 19 g, indicated for small-to-medium-sized dogs and 25 g for large-sized dogs, with 0.760 g and 1 g, respectively, of the active ingredient. The total study period was 30 months.

*Capture points* - We selected phlebotomine fly capture points, or dwellings, in four neighbourhoods in each municipality (Figs 1 and 2), with 10 and 12 capture points chosen in Montes Claros and Fortaleza, respectively. In the first municipality (Montes Claros), five capture points were in the intervention area, and five were in the control area. In Fortaleza, nine capture points were in the intervention area, and three were in the control area. We chose capture points (domiciles) with a minimum distance of 200-500 metres between them. The domiciles were chosen according to characteristics indicative of their receptiveness to the vector: type of vegetation present, presence of domestic animals, accumulation of organic matter, and peridomestic area (200-300 square metres). Another criterion for the selection of points was the presence of dogs. A dog resided at each phlebotomine fly capture point.

*Phlebotomine fly collection* - We collected phlebotomine flies using CDC light traps, which were set three consecutive nights per month, from 6 pm to 6 am for 30 months (MS/SVS/DVE 2014). Two traps were placed in each residence, one inside of the house and one outside, preferably near places where dogs sheltered (MS/SVS/DVE 2014). Fly capture began five months after the first placement of collars in Montes Claros and Fortaleza.

*Phlebotomine fly identification* - Flies captured in Montes Claros were sent to the Entomology Laboratory of the Zoonosis Control Centre of the municipality, where male flies were screened and identified. The females were sent to the Medical Parasitology and Vector Biology Laboratory of the University of Brasilia for slide preparation and identification in accordance with the Galati (2003) classification. Flies captured in Fortaleza were taken to the Dr Thomaz Correia Aragão Medical Entomology Laboratory of the state of Ceará Health Department, where males and females were identified in accordance with the Young and Duncan (1994) classification. Both taxonomic keys are recommended by the Ministry of Health; therefore, the choice of which key to adopt in the daily routine was the decision of entomology teams in each state. There was no distinction in the characteristics used to identify *Lu. longipalpis* between the taxonomic keys.

*Data analysis* - Formulas shown in Table I were used to calculate the domicile infestation and distribution rates of *Lu. longipalpis* at each capture site (MS/SVS/DVE 2014). A descriptive analysis of domicile infestation and relative vector abundance in intervention and control areas was performed. We used a locally-weighted scatterplot smoothing technique (“lowess”) to describe the variation in sandflies captured across time considering the study area (Fortaleza, CE, and Montes Claros, MG), intervention area [with deltamethrin-impregnated collars and without (control)], and capture site (inside and outside of houses). The “lowess” smoother is a non-parametric regression method that fits a locally weighted linear regression model giving points closer to each value the greater weight in smoothing and limiting the effect of outliers. To determine associations between intervention, capture site, and insect abundance according to study site and period of temporal aggregation (bimester), we used a Poisson regression model. In Poisson regression, associations are expressed as incidence rate ratios (IRR) with 95% confidence intervals (95% CI). For the data analyses, we used STATA version 12.0.

**TABLE I:** Formulas used to calculate the domicile and relative vector abundance rate

Domicile infestation rate	Number of positive domicile/researched site/technique
	Number of researched domicile
Relative abundance	Number of *Lutzomyia longipalpis* collected per methodology in the domiciles (intra or peridomicile)
	Number of researched domicile

## RESULTS

In total, 4,373 (1,494 intradomiciliary and 2,879 peridomiciliary) and 40,797 (8,359 intradomiciliary and 32,438 peridomiciliary) specimens of *Lu. longipalpis* were collected in Fortaleza and Montes Claros, respectively.

Out of the 4,373 phlebotomine flies captured in Fortaleza, 3,141 were males and 1,232 were females, and, out of the 40,792 captured in Montes Claros, 36,716 were males and 4,076 females. From Montes Claros, 310 specimens were lost because of poor quality or the protocol used for the identification, corresponding to 7.6% of all female specimens from this municipality. When compared in absolute numbers, this loss was similar in both the intervention and control areas.

**Fig. 3:**
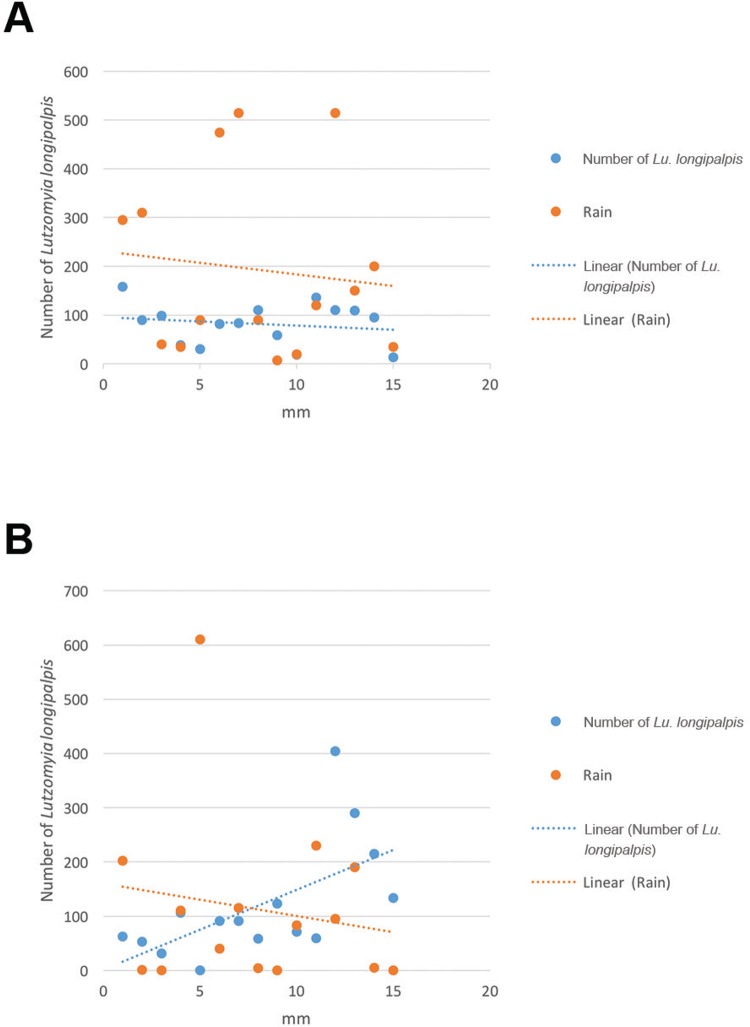
: number of *Lutzomyia longipalpis* specimens captured per bimester and observed rainfall during the study period. (A) Fortaleza, Ceará (CE), Brazil. (B) Montes Claros, Minas Gerais (MG), Brazil, from 2013 to 2015.

In the full study period, the infestation rate was 100% in both municipalities, confirming the wide distribution of *Lu. longipalpis* in all four neighbourhoods. However, there were differences in relative abundance. In the control area in Fortaleza, the relative abundance of *Lu. longipalpis* across the study period was 415 specimens per capture point, whereas in the intervention area it was 159.25. The relative abundances inside of houses were 135.88 and 67.75 and outside of houses were 279.22 and 91.5 for the control and intervention areas, respectively. Vector densities varied throughout the study period, with an increase in *Lu. longipalpis* captured during the rainy season or soon after (Fig. 3). The overall male:female ratio was 2.54:1, with the ratio higher in the intervention areas (3.1:1) than in the control areas (2.4:1). Across the capture period in the control area in Montes Claros, the relative abundance of *Lu. longipalpis* was 5,660 specimens per capture point, whereas in the intervention area it was 2,499.4. The relative abundances of *Lu. longipalpis* inside of houses were 964.4 and 707.4 and outside of houses were 4,695.6 and 1,792 for the control and intervention areas, respectively. Vector densities varied throughout the study period, with an increase in *Lu. longipalpis* captured during the rainy season or soon after (Fig. 3). The overall male:female ratio was 9.07:1, with the ratio higher in the intervention areas (12.03:1) than in the control areas (8.15:1).

In the 30-month study period, Fortaleza had a total of 2,897 mm of rain, with rainfall in the peak periods ranging from 400 to 500 mm in March to July of the study years. In the 9th bimester, only 7 mm of rainfall was recorded. In contrast, Montes Claros had 1,685 mm of rainfall, with a homogeneous distribution throughout the year and a single registered peak in November and December 2013. On the 3rd, 9th, and 15th bimesters, it did not rain (Fig. 3).

**Fig. 4:**
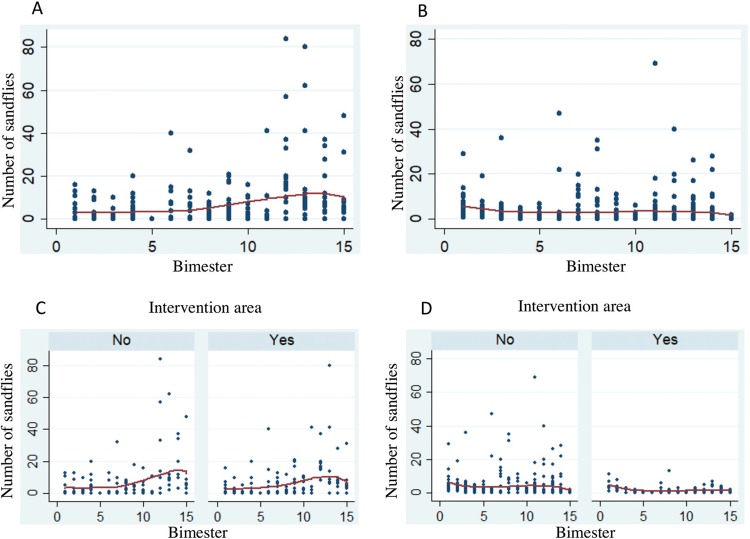
: total number of *Lutzomyia longipalpis* captured per bimester in the intervention area (with and without the use of dog collars) in Montes Claros, Minas Gerais (MG), and Fortaleza, Ceará (CE), Brazil, from 2013 to 2015. (A) Number of *Lu. longipalpis* captured per bimester, Montes Claros, MG. (B) Number of *Lu. longipalpis* captured per bimester, Fortaleza, CE. (C) Number of *Lu. longipalpis* captured per bimester in intervention and control areas, Montes Claros, MG. (D) Number of *Lu. longipalpis* captured per bimester in intervention and control areas, Fortaleza, CE.

**Fig. 5:**
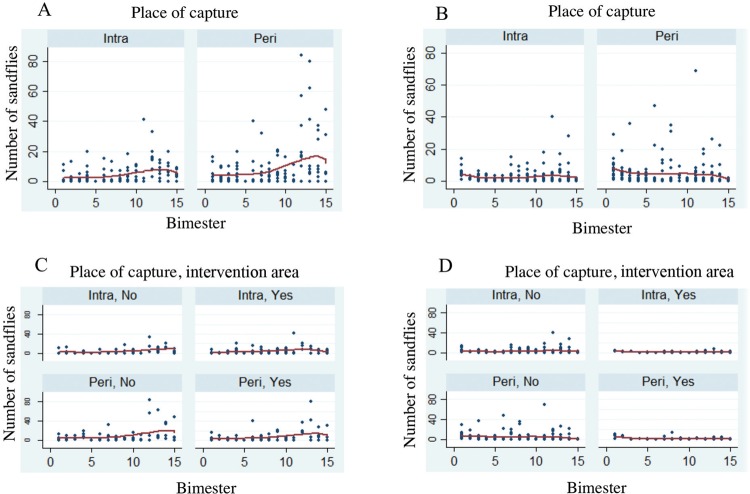
: number of *Lutzomyia longipalpis* captured per bimester at each collection site (intra- and peridomiciliary) and intervention or control area (areas with and without the use of dog collars) in Montes Claros, Minas Gerais (MG), and Fortaleza, Ceará (CE), Brazil. (A) Number of *Lu. longipalpis* captured per bimester and at each capture site in Montes Claros, MG. (B) Number of *Lu. longipalpis* captured per bimester and at each capture site in Fortaleza, CE. (C) Number of *Lu. longipalpis* captured per bimester and at each capture site and intervention or control area, Montes Claros, MG. (D) Number of *Lu. longipalpis* captured per bimester and at each capture site and intervention or control area, Fortaleza, CE.

The use of dog collars was associated with 15% (p = 0.004) and 60% (p < 0.001) reductions in the number of captured insects in Montes Claros and Fortaleza, respectively (Tables II-III). The analysis according to capture site showed a 21% decrease in peridomiciliary *Lu. longipalpis* (IRR = 0.783; p < 0.001) in Montes Claros and a 56% (IRR = 0.44; p < 0.001) and 60% (IRR = 0.40; p < 0.001) decrease in intra- and peridomiciliary *Lu. longipalpis* in Fortaleza, respectively (Fig. 5) (Tables II-III). No seasonality or other cyclical phenomena were observed in either city (Fig. 4).

**TABLE II:** Poisson regression model. Evaluation of the association between the intervention, capture site and insect abundance, according to the period of temporal aggregation (bimester), Montes Claros, Minas Gerias (MG), Brazil

Total_insects	IRR	P > |z|	95% confidence interval	
Time	1.14	0.000	1.12	1.15
1.collar	.87	0.004	.79	.95
2.local	1.92	0.000	1.74	2.12
_cons	1.45	0.000	1.24	1.69

**TABLE III:** Poisson regression model. Evaluation of the association between the intervention, capture site and insect abundance, according to the period of temporal aggregation (bimester), Fortaleza, Ceará (CE), Brazil

Total_insects	IRR	P>|z|	95% confidence interval	
Time	.97	0.002	.96	.99
1.collar	.41	0.000	.35	.49
2.local	1.79	0.000	1.59	2.01
_cons	3.37	0.000	2.94	3.87

## DISCUSSION

The 4% deltamethrin-impregnated collar is currently considered a tool for VL control. Some studies have indicated that its use in transmission areas reduces the prevalence of canine VL, but these studies have not shown its effects on the vector population (Kazimoto 2016, Sevá et al. 2016). This effect on the prevalence of canine VL is based on a reduction in blood feeding by the vector on dogs with collars, mediated by the repellent and insecticidal action of deltamethrin, a pyrethroid, impregnated in the collars (Halbig et al. 2000, David et al. 2001, Maroli et al. 2001). However, one study showed a decrease in *Lu. longipalpis* mortality, from 90% to 35% between the 4th and 37th week of collar use, demonstrating the need for systematic reapplications of the insecticide for continued interruption of vector feeding on dogs (David et al. 2001).

Scalibor® collars were first changed after six months in the intervention areas, based on the manufacturer’s specification. However, between the 1st and 2nd exchanges, the interval was extended to one year because of problems with product delivery. Although not quantified, there were losses of the collars throughout the study for several reasons: removal by dog owners because of itching, irritation, and dermatitis in dogs that wore the collars; fights between animals; removal of collar by the animals, mainly by semi-domiciled dogs, because most were not accustomed to wearing collars; and other reasons. Reithinger et al. (2004), using a mathematical model, demonstrated a reduction in antibody seroconversion by 50% in animals given collars, despite 41% collar losses predicted during the study. In the present study, even with losses, a 40% reduction in canine prevalence, as well as a reduction in *Lu. longipalpis* inside and outside of houses, was observed in Fortaleza and Montes Claros (unpublished observations, Werneck 2016). The effect on canine prevalence and reduction in *Lu. longipalpis* can be attributed to the herd effect of collar use by more than 50% of dogs.

This study showed a wide distribution of the main vector species for VL, *Lu. longipalpis*, in both municipalities, confirming the ability of this species to adapt to an anthropised environment. Adaptation of *Lu. longipalpis* to urbanised areas in these two municipalities was described previously, and this ability to exploit an urban ecology underlies the high correlation between human cases and canine seroprevalence (Michalsky et al. 2009, Silva et al. 2014).

At collection sites, we observed a higher abundance of *Lu. longipalpis* outside of houses than within them in both municipalities. This can be explained by characteristics favourable to the vector in these locations: peridomestic areas with fruit trees and more than one type of domestic animal or livestock, meaning that the phlebotomine flies did not have to enter houses to search for food (Silva et al. 2014, Salomón et al. 2015).

The number of *Lu. longipalpis* specimens captured in Montes Claros was ten-fold the number captured in Fortaleza. The number of *Lu. longipalpis* specimens captured outside of houses was four times more than that of specimens capture inside houses in Montes Claros; two times more specimens were captured outside than inside of houses in Fortaleza. These differences can be attributed to the fact that Montes Claros is a city with a large amount of green space and intense urbanisation with frequent environmental modifications. As previously described, urbanisation contributes to the destruction of natural habitats for the *L. infantum* vector and reservoirs, causing the vectors to enter anthropised environments and facilitating interactions between the vector and humans (Cardim et al. 2013). Furthermore, the high degree of anthropophilia and eclectic food preferences of phlebotomine fly species may allow them to adapt to various anthropised environments (Salomón et al. 2015). Because the adaptation of sandflies to anthropic environments occurs gradually, sandflies are still found primarily in areas similar to their natural habitat (peridomiciliary spaces) and may later adapt to more anthropised areas such as the inside of houses (Silva et al. 2014).

Likewise, although the environmental characteristics of the capture points were similar in each municipality surveyed, there were some differences between them that could account for the difference in *Lu. longipalpis* densities between municipalities. Unlike what was observed in Fortaleza, in Montes Claros, poultry, especially chickens, which are associated with the presence phlebotomine flies, were found in all capture sites (Alexander et al. 2002, Afonso et al. 2012, Soares et al. 2013). Moreover, the presence of poultry may act as an amplifier for these flies (Soares et al. 2013). Studies carried out in the Posadas province of Argentina showed a correlation between the presence of birds and absence of electric power and an increase in the density of *Lu. longipalpis* (Fernández et al. 2010).

Additionally, there were differences in rainfall. Rainfall at moderate levels is associated with an increase in phlebotomine flies; rainfall at elevated levels, however, can destroy *Lutzomyia* breeding sites, decreasing the population. In Fortaleza, we observed that the number of captured specimens was larger in the period soon after the peak in rainfall, which agrees with previous reports (Michalshy et al. 2009). However, in Montes Claros, this was not the case, with an absence of peaks in rainfall and presence of phlebotomine flies throughout the study period. This consistency in the number of specimens captured, regardless of the month, has been described previously (Michalsky et al. 2009).

The use of 4% deltamethrin-impregnated collars resulted in 14% and 60% reductions in *Lu. longipalpis* captured in the intervention areas compared to the control areas in Montes Claros and Fortaleza, respectively. This reduction is in line with the 40% decrease in canine prevalence observed in municipalities that participated in the project entitled “Evaluation of the effectiveness of 4% deltamethrin-impregnated collars in endemic areas for visceral leishmaniasis” (unpublished observations, Werneck 2016). Even though the natural infection rate in captured specimens is not routinely determined by the health services, the number of captured insects, in associated with the high degree of anthropophilia of this species and their proximity to domestic reservoirs, may indicate a higher risk for transmission of *L. infantum* (Salomón et al. 2015).

Regarding the ratio of male to female *Lu. longipalpis*, in both municipalities and areas (intervention and control) the number of captured males was higher than that of females. This predominance of males has been described before (Cabanillas and Castellón 1999, Silva et al. 2014). Some authors have suggested that the use of light traps may cause an imbalance in the sexes captured, with males caught more frequently than females. This could be a result of the aggregation of male for copulation with females (de Aguiar et al. 1985).

In both the intervention and control areas in both municipalities, the *Lu. longipalpis* infestation rate was 100%, indicating that the presence of the dog collar, despite reducing the number of species captured, did not prevent houses from becoming infested. Nevertheless, because of the repellent and insecticidal effects of the collar, the vector may not access blood meals from dogs, which are the main source of *L. infantum* in urban areas, and therefore there are not as likely to become infected and transmit the pathogen (Lainson and Rangel 2005, Maroli et al. 2013).

In addition, it is important to evaluate the food sources selected by *Lu. longipalpis* when dogs are unavailable for feeding. At each capture point in both the intervention and control areas, there was at least one dog and another animal as a food source, in addition to humans. Understanding feeding preferences would help in assessing the risk of transmission of *L. infantum* to humans. Protection of dogs by the collars may increase interactions between *Lu. longipalpis* and humans, which could increase the number of VL cases in humans. However, this increase would not be sustained in the long term because of the sources of infection for the vector would be reduced. Studies have shown that *Lu. longipalpis* feeds eclectically; however, it has a preference for birds, followed by pigs, dogs, and then humans (Afonso et al. 2012).

In the intervention area of Montes Claros, the number of *Lu. longipalpis* captured outside of houses was reduced by approximately 21% over the study period (p < 0.001), with no decrease inside of houses. In Fortaleza, the reduction was more pronounced both inside and outside of houses, with 56% and 60% reductions, respectively (p < 0.001). This reduction outside of houses was expected, because *Lu. longipalpis* would feed less on dogs with collars and resort to feeding on other domestic animals, livestock, or humans.

Although identifying factors associated with the maintenance of VL, mainly those related to the presence of *Lu. longipalpis* in the area, reducing the population of this vector indisputably decreases the probability of *L. infantum* transmission and thus cases of the disease. The results of this work indicate that use of 4% deltamethrin-impregnated collars should be integrated into the roster of activities currently recommended by the Ministry of Health for VL control, because current control strategies in urban areas, such as euthanising domestic reservoirs, are unpopular. An evaluation of *L. infantum* infection rates and food sources of captured females may complement this data, further validating the effects of this intervention on the prevalence of VL.
